# Candidate gene resequencing to identify rare, pedigree-specific variants influencing healthy aging phenotypes in the long life family study

**DOI:** 10.1186/s12877-016-0253-y

**Published:** 2016-04-09

**Authors:** Todd E. Druley, Lihua Wang, Shiow J. Lin, Joseph H. Lee, Qunyuan Zhang, E. Warwick Daw, Haley J. Abel, Sara E. Chasnoff, Enrique I. Ramos, Benjamin T. Levinson, Bharat Thyagarajan, Anne B. Newman, Kaare Christensen, Richard Mayeux, Michael A. Province

**Affiliations:** Center for Genome Sciences and Systems Biology, Washington University School of Medicine, 660 South Euclid Avenue, Campus Box 8116, St. Louis, MO 63108 USA; Department of Pediatrics, Washington University School of Medicine, 660 South Euclid Avenue, Campus Box 8116, St. Louis, MO 63108 USA; Division of Statistical Genomics, Department of Genetics, Washington University School of Medicine, St. Louis, MO USA; Sergievsky Center, College of Physicians and Surgeons, Columbia University New York, New York, NY USA; Taub Institute, College of Physicians and Surgeons, Columbia University New York, New York, NY USA; Department of Epidemiology, School of Public Health, Columbia University New York, New York, NY USA; Department of Laboratory Medicine and Pathology, University of Minnesota, Minneapolis, MN USA; Department of Epidemiology, University of Pittsburgh Graduate School of Public Health, Pittsburgh, PA USA; The Danish Aging Research Center, Epidemiology, University of Southern Denmark, Odense, Denmark; Gertrude H. Sergievsky Center and the Taub Institute for Research on Alzheimer’s Disease and the Aging Brain, Columbia University, New York City, NY USA

**Keywords:** Genomics, Aging, Genetics, Geriatrics, Pedigrees, Family, Sequencing

## Abstract

**Background:**

The Long Life Family Study (LLFS) is an international study to identify the genetic components of various healthy aging phenotypes. We hypothesized that pedigree-specific rare variants at longevity-associated genes could have a similar functional impact on healthy phenotypes.

**Methods:**

We performed custom hybridization capture sequencing to identify the functional variants in 464 candidate genes for longevity or the major diseases of aging in 615 pedigrees (4,953 individuals) from the LLFS, using a multiplexed, custom hybridization capture. Variants were analyzed individually or as a group across an entire gene for association to aging phenotypes using family based tests.

**Results:**

We found significant associations to three genes and nine single variants. Most notably, we found a novel variant significantly associated with exceptional survival in the 3’ UTR *OBFC1* in 13 individuals from six pedigrees. *OBFC1* (chromosome 10) is involved in telomere maintenance, and falls within a linkage peak recently reported from an analysis of telomere length in LLFS families. Two different algorithms for single gene associations identified three genes with an enrichment of variation that was significantly associated with three phenotypes (*GSK3B* with the Healthy Aging Index, *NOTCH1* with diastolic blood pressure and *TP53* with serum HDL).

**Conclusions:**

Sequencing analysis of family-based associations for age-related phenotypes can identify rare or novel variants.

**Electronic supplementary material:**

The online version of this article (doi:10.1186/s12877-016-0253-y) contains supplementary material, which is available to authorized users.

## Background

According to United Nations World Population Prospects 2012 revision (http://esa.un.org/unpd/wpp/Documentation/pdf/WPP2012_HIGHLIGHTS.pdf), the worldwide average human lifespan was 71 years (68.5 years for males and 73.5 years for females) over the period 2010–2013. At least 25 % of human lifespan is dictated by genetic factors, most of which is unknown [1]. Currently, most human longevity and healthy aging related variants identified through genome wide association study (GWAS) are either intergenic or intronic with weak effects, and there is little characterization of coding variants that may influence human lifespan. For instance, common variants in multiple genes such as apolipoprotein E (*APOE*) [2–5], Forkhead Box O1 (*FOXO1*) [6], Forkhead Box O3 (*FOXO3*) [7], Insulin-Like Growth Factor 1 Receptor (*IGF-1R*) [6, 8] and Translocase of Outer Mitochondrial Membrane 40 Homolog (*TOMM40*) [9] have all been associated with human lifespan. However, lifespan is a highly complex trait and healthy aging is controlled and influenced by variety of environmental factors and individual phenotypes [10]. Leukocyte telomere length, Body Mass Index (BMI), blood pressure, serum lipids, blood glucose, cognitive function, onset of type 2 diabetes, heart disease, cancer, and stroke are a few phenotypes associated with aging, and we hypothesized that pedigree-specific coding variants associated with these traits would identify additional genes or pathways important for regulating human lifespan. While non-coding variants may have significant gene or epigenetic regulatory effects, exonic variants would be expected to exert larger effects on gene function and might therefore explain a larger fraction of the variance in these complex aging phenotypes.

Thus, we expect families clustered for long-lived healthy members [11, 12] to be enriched for genetic variants that will promote healthy and/or prolonged aging. To identify new, rare, heritable variants that are associated with multiple healthy aging phenotypes, we performed custom hybridization capture sequencing of 464 healthy aging related candidate genes in Phase 1 of the Long Life Family Study (LLFS). We then performed association analyses of both rare (<1 % minor allele frequency) and common variants against multiple aging-related phenotypes including survival, leukocyte telomere length, healthy aging index [13], BMI, blood pressure, blood lipid levels, blood glucose level, cognitive function, onset of type 2 diabetes, heart disease, cancer, and stroke.

## Methods

### Subjects

The LLFS [11], a multi-center family-based cohort study, enrolled 4,953 individuals from 539 families clustered for exceptional survival to identify environmental and genetic factors that account for the long healthy lives in these families. Phase 1 of the LLFS was conducted between 2006 and 2009. All participants provided written informed consent prior to inclusion in the study. The four recruitment centers include Boston University Medical Center, Columbia University Medical Center, University of Pittsburgh, and University of Southern Denmark. Using the Family Longevity Selection Score (FLoSS) [14], a score generated according to birth-year cohort survival probabilities of the proband and siblings, probands and their families with FLoSS score of 7 or higher, at least one living sibling, and at least one living offspring (minimum family size of 3), who were able to give informed consent and willing to participate were recruited. The spouses were enrolled as controls for this study. The characteristics of the LLFS population by generation and by gender are listed in Additional file 1: Table S1 and S2, respectively.

### Phenotype measurements

In this study, human lifespan and phenotypic traits related to healthy aging including survival, leukocyte telomere length, BMI, blood pressure, blood lipid levels, blood glucose and insulin levels, cognitive function, healthy aging index, as well as the ages of onset of stroke, coronary heart disease, congestive heart failure, cancer, and type 2 diabetes mellitus, were investigated.

### Survival and mortality-weighted healthy aging index

The lifespan in LLFS was estimated as the duration of survival free of death from any cause or from any one of a specified list of diseases including cardiovascular disease, congestive heart failure, cancer, stroke, and Type 2 diabetes. The LLFS proband generation, including probands and full and half siblings (*n* = 810), was used for identifying variants related with survival.

Healthy aging was established by the lack of disease and clinical test values indicating normal function of various biological systems. To increase the probability of identifying healthy aging-related genetic variants, which might influence one or more components of these biological systems, Sanders et al. proposed the Healthy Aging Index (HAI) as a subphenotype of longevity [13]. Using approximate age-adjusted tertiles for systolic blood pressure, pulmonary vital capacity, creatinine, fasting glucose, and Modified Mini-Mental Status Examination score, each individual is scored 0, 1, or 2 for each trait. The HAI is then calculated from the sum of these scores in a range from 0 (healthy) to 10 (unhealthy). The mortality-weighted HAI is generated from the sum of the mortality-weighted scores of the five components listed above. The weights are based on the regression coefficients from the Cox proportional hazards model for the effect of each component on survival and are 0.17085 for systolic blood pressure, 0.38386 for forced vital capacity, 0.42873 for MMSE points, 0.13397 for serum creatinine, and 0.23880 for serum fasting glucose.

### Glycated hemoglobin (HbA_1c_), Blood Glucose and Insulin Levels, Leukocyte Telomere Length, Blood Lipid Levels Including Total Cholesterol, HDL Cholesterol, LDL Cholesterol and Triglyceride, BMI, Blood Pressure, Cognitive Function, Stroke, Coronary Heart Disease, Congestive Heart Failure, Cancer, Type 2 Diabetes

We looked for any possible association between our candidate genes related to healthy aging and various aging phenotypes including HbA_1c_, blood glucose and insulin levels, leukocyte telomere length, blood lipid levels including total cholesterol, HDL cholesterol, LDL cholesterol and triglyceride, BMI, blood pressure, cognitive function, stroke, coronary heart disease, congestive heart failure, cancer, and type 2 diabetes.

As described previously [15], blood samples were collected between 2006 and 2009, and HbA_1c_ was measured using identical ion exchange-based high performance liquid chromatography with the Tosoh 2.2 Plus and after 2007 with the Tosch G7 Glycohemoglobin Analyzer (Tosoh Medics, San Francisco, CA 94080) at the Advanced Research and Diagnostics Laboratory, University of Minnesota. Fasting glucose was measured after an 8-h fast in serum by the Roche hexokinase method (Roche Diagnostics, Indianapolis, IN 46250) on a Roche Modular P Chemistry Analyzer (Roche Diagnostics Corporation). Fasting insulin was measured after an 8-h fast in serum on a Roche Elecsys 2010 Analyzer (Roche Diagnostics Corporation) using a sandwich immunoassay method (Roche Diagnostics, Indianapolis, IN 46250). Assays of average leukocyte telomere length were described by Lee et al. [16]. Briefly, Telomeres (T) and beta-globin control (S) were amplified using real-time PCR of 95 °C for 10 min denaturation, 34 cycles of 95 °C for 15 s and 55 °C for 120 s performed on the CFX384 thermocycler (BioRad, Richmond, CA). The leukocyte telomere length was calculated from T/S ratio using the linear regression formula of *bp* = (1,585 ∗ T/S ratio) + 3582. Fasting total cholesterol was measured after an 8-h fast in serum using a cholesterol oxidase method (Roche Diagnostics, Indianapolis, IN 46250) on a Roche Modular P Chemistry Analyzer.

Fasting HDL-cholesterol was measured after an 8-h fast directly in serum using the Roche HDL-Cholesterol 3rd generation direct method (Roche Diagnostics, Indianapolis, IN 46250) on a Roche Modular P Chemistry Analyzer. Fasting triglycerides were measured after an 8-h fast in serum using Triglyceride GB reagent (Roche Diagnostics, Indianapolis, IN 46250) on a Roche Modular P Chemistry Analyzer. LDL-cholesterol was calculated by the Friedewald equation using the measured results for total cholesterol, HDL-cholesterol, and triglycerides. This equation (LDL-cholesterol = total cholesterol – HDL – (triglycerides/5)) can be used to calculate LDL-cholesterol when triglycerides are less than 400 mg/dL. The corrected values for triglyceride and LDL cholesterol was calculated for individuals taking lipid lowering medications [17]. BMI was calculated as weight (kg)/height (m)^2^. Sitting systolic and diastolic blood pressure was obtained by the average of three measures using an automated blood pressure machine (BP-tru BPM 300, VMS MedTech, Coquitlam, Canada). Pulse Pressure was calculated as systolic blood pressure minus diastolic blood pressure. These results were corrected for individuals taking blood pressure modifying medication as previously published [18]. General cognitive function including arithmetic, memory, and orientation was assessed using the mini-mental state examination (MMSE) or Folstein test [19]. Stroke or cerebrovascular accident includes self-reported stroke, transient ischemic attack (TIA), or mini-stroke. Coronary heart disease was defined as self-reported myocardial infarction, heart attack, coronary angioplasty, or coronary artery bypass grafting. Heart failure or congestive heart failure and cancer (including breast cancer, colon or rectal cancer, esophageal cancer, leukemia or lymphoma, lung cancer, pancreatic cancer, prostate cancer, etc.) were self-reported. Type 2 diabetes was defined as use of diabetes medications or fasting glucose ≥ 126 mg/dl.

### Genome-wide SNP array genotyping

Illumina Human Omni 2.5 v1 was used to perform whole genome SNP genotyping on each study participant by CIDR (www.cidr.jhmi.edu), and the resulting data has been submitted to dbGaP under accession number phs000397.v1.p1. Genome-wide SNP variants within targeted sequenced regions were used as controls for sequencing accuracy as described below.

### Pooled sequencing and indexed custom library preparation

We have previously published the entire protocol for custom hybridization capture of multiplexed, indexed next generation sequencing [20, 27]. Briefly, customized adapter, blocker, pre- and post-hybridization PCR amplification primers and all index sequences used are listed in Additional file 1: Table S5 of the same report. We performed pooled capture of 464 candidate genes (Additional file 1: Table S3) selected collectively by the LLFS investigators due to their published association with age-related phenotypes. Candidate gene resequencing was performed from individually indexed DNA samples from LLFS participants [https://longlifefamilystudy.wustl.edu/LLFS/Home.html]. The bait set was created using the Agilent eArray online tool [https://earray.chem.agilent.com/earray/] for the Agilent SureSelect Custom DNA Capture [http://www.genomics.agilent.com] using 2X tiling, an exon-centered layout strategy, and eliminating probes that overlap standard repeat masked regions by 20 or more bases. In total, there were 2,500,709 bases (2.5 Mb) covered by baits in 6,966 distinct intervals.

### Sequencing

Sequencing was performed in the Genome Technology Access Center at Washington University using the HiSeq 2000 platform, generating 101 bp paired-end reads. GWA and sequencing information for the LLFS participants is available in dbGaP using accession number phs000397.v1.p1.

### Sequencing alignment, variant calling and filtering

Sequencing analysis for indexed captures of all LLFS participants followed previously reported methods (27; section entitled “Pooled indexed custom capture data analysis” for more details). Briefly, raw sequence data was aligned against the human genome (hg19/NCBI 37.0) using Novoalign (Novocraft, Inc.) and, from aligned reads, variants were called using samtools-0.1.18 mpileup at only the 2.5 Mb of target sequence within our custom hybridization array.

The thresholds used for filtering called variants from sequencing data were determined by comparing common base positions shared with existing GWA data. For gene-level burden tests, variants were filtered for coverage <5-fold, quality score <45, call rate < 60 %, MAF > 0.01, Mendel errors, and nonfunctional variants. Single variants were filtered for coverage <5-fold, quality score <45, call rate < 60 %, and Mendel errors. For rare variants (<2 % minor allele frequency), these thresholds provided a sensitivity of ≥94.9 % and specificity of ≥99.9 % [27]. Mendel error cutoffs were implemented as follows: MAF = 0 to <0.01 for >2 pedigrees with the Mendel error; MAF = 0.01 to <0.05 for ≥7 pedigrees; MAF = 0.05 to <0.1 for ≥12 pedigrees; MAF = 0.1 to <0.2 for ≥26 pedigrees; MAF = 0.2 to <0.3 for ≥30 pedigrees; MAF = 0.3 to <0.4 for ≥32 pedigrees; MAF = 0.4 to <0.51 for ≥38 pedigrees.

### Statistical analyses

#### Trait exceptionality scores for survival, BMI, blood pressure, blood lipid levels, blood glucose level, blood insulin level, cognitive function, type 2 diabetes, heart disease, cancer, and stroke

The LLFS cohort is enriched with longer-lived and healthier individuals than the general population, as well as the Framingham Heart Study. To account for this difference and increase our statistical power for detecting sequence variants associated with aging-related phenotypes, such as survival, BMI, blood pressure, blood lipid levels, blood glucose level, blood insulin level, cognitive function, type 2 diabetes, heart disease, cancer, and stroke are transformed to “trait exceptionality scores”. Higher trait exceptionality scores indicate that, for a given trait, an individual is significantly different from the reference population for the same trait, resulting in longer individual survival and/or better health. Using sex- and birth-year specific cohort life tables from the 2012 trustees report of the United States Social Security Administration, the trait exceptionality scores for survival were calculated for the oldest (proband) generation in LLFS, along with any blood relatives within the same generation regardless of age. These calculations are conditional on survival to age 40 and were determined as the negative logarithm of the probability of survival beyond age at last contact. Trait exceptionality scores for BMI, blood pressure, blood lipid levels, blood glucose level, blood insulin level, and cognitive function were also calculated as the negative logarithm of the *probability of the trait score* compared to the age/sex/birth-cohort matched in the Framingham Heart Study. Trait exceptionality scores for type 2 diabetes, heart disease, cancer, and stroke were calculated as the negative logarithm of the *probability of the onset age of the disease*, and compared to the distribution of the age of onset for each trait in the Framingham Heart Study. There are two additional components to the HAI, which are pulmonary vital capacity and serum creatinine. However, these were not available in Framingham Heart Study and were not included in our analyses.

#### Phenotype transformation and covariates adjustment

To increase the power and reduce the confounder effects of our analyses, we performed the following covariates adjustment and transformation. HbA_1c_ was adjusted for age, age^2^, age^3^, field center and the top twenty principal components (PCs); the standardized residuals from a stepwise covariate adjustment were used as final phenotype for the following association analyses. The leukocyte telomere length was transformed using an inverse normal function. This transformed trait was adjusted for covariates age, sex, education, field center, smoking, alcohol consumption, marital status, history of heart disease, and twenty PCs (PC8 was the only significant PC associated with leukocyte telomere length in the multivariate polygenic model). Mortality weighted healthy aging index were adjusted for age, sex, and 10 PCs and the residuals were used in the following analyses. The residuals of trait exceptionality scores adjusted for field centers and 20 PCs were used in the following analyses.

#### Single variant association testing

Family relatedness was estimated based on pedigree structure using the “kinship” R package. Accounting for this relatedness within families as random effects, the additive genetic fixed effects of SNPs were analyzed using the linear mixed effects model implemented in the “lmekin” R packages [21, 22]. Q-Q plots for the phenotypes analyzed are shown in Additional file 1: Figure S1 and demonstrate that, compared to genomic control (calculated as median of observed p value / median of expected p value to avoid estimation bias), each is within 0.578–1.09 using this approach. Thus, the type I error rate is low in this study.

#### Rare variant testing across genes

Currently un-weighted sum score (UWSS) [23], weighted sum score (WSS) [24], P-value Weighted Sum Test (PWST) method [25] and family based sequence kernel association test (famSKAT) [26] are commonly used for estimating the effects of rare variants. Because the PWST and famSKAT are likely to have greater power to detect rare causal variants, we applied these two algorithms to assess the influence of called rare variants. This was necessary since standard association tests of individual rare variants (MAF < 0.01) are underpowered unless sample sizes or effect sizes are very large, functional variants within the same gene based on bioinformatics annotation by ANNOVAR [27] were analyzed together in this study. For each group of variants, UWSS was calculated as the total number of variant alleles carried by a subject. The weight was calculated based on allele frequency in controls and WSS was calculated as the weighted sum of minor alleles over a group of variants. The UWSS or WSS score was treated as a single predictor variable (*X*) and fit into a linear model. The kinship matrix was incorporated into the linear models to adjust for familial relatedness between subjects. Parameters in each model were estimated by the maximum likelihood algorithm and tested by the Wald test. Rather than using a fixed weight, the PWST method adaptively calculates the weights for individual variants from the observed genotype and phenotype data and then performs a permutation procedure for family data to avoid false positive inflation due to the over fitting of the adaptive score WSS.

## Results

### Sample characteristics

#### Phenotype

Our results included 4,217 LLFS subjects (mean of age 70.56 for men and 70.05 for women) with complete phenotypic and genotypic information. Individuals with inadequate GWA or sequencing coverage were excluded. The study participants tend to have healthier profiles compared with other cohorts of adults. For these individuals, the mean score of healthy aging index (3.64 in men and women) and mortality weighted healthy aging index (3.24 in men and 3.03 in women) are low. The average age of the LLFS probands and their relatives (95.8 in men and 99.4 in women) is much higher than the cohort life expectancy of US and Danish (an LLFS study site) early 20^th^ century birth cohorts (60–70 years) and the current life expectancy in both populations (~80 years).

#### Sequencing results

**Fig. 1:**
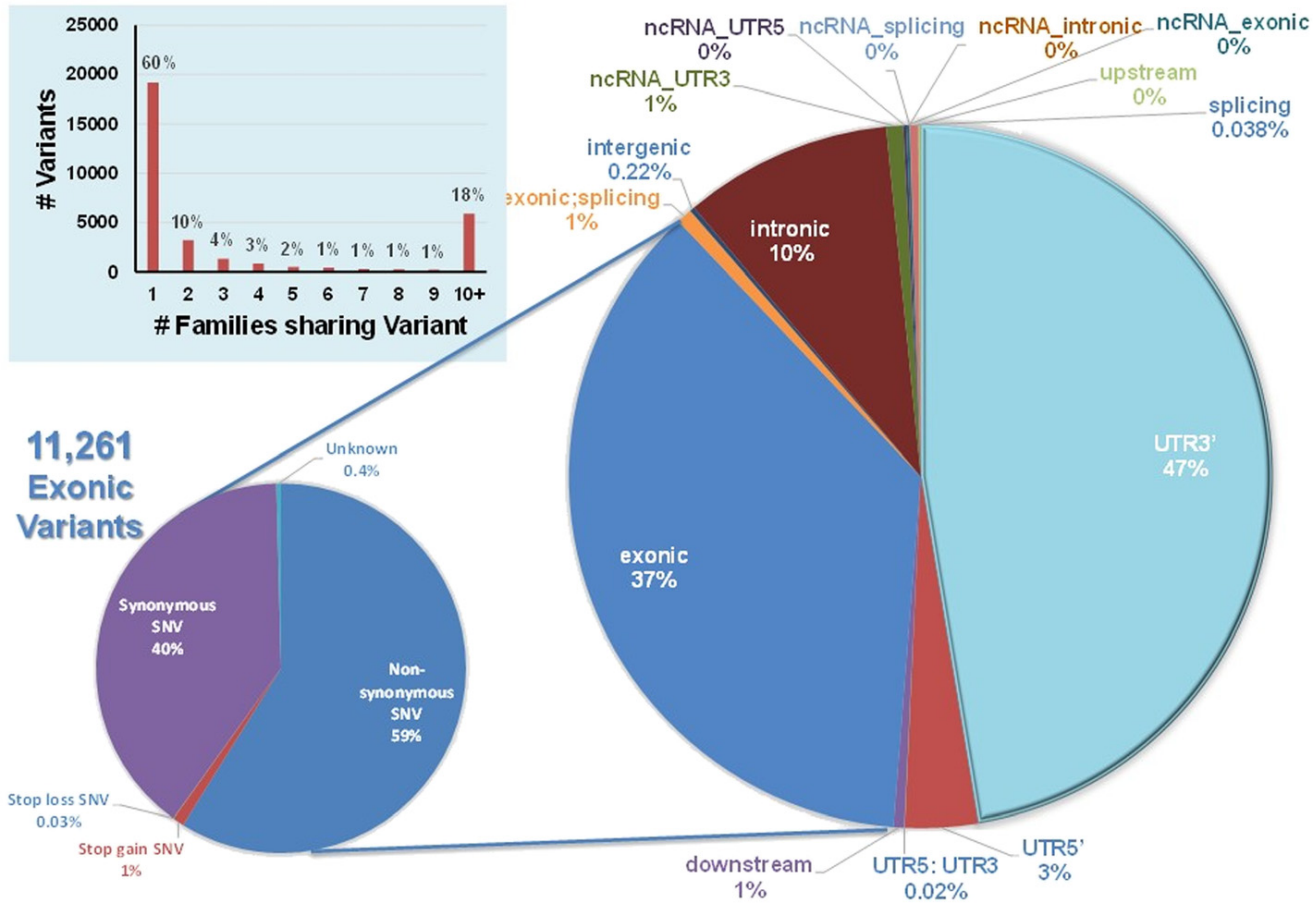
The distribution of sequenced variants within different genomic region

To reduce false positive variant calls, stringent filtering of raw sequence data was employed and is described in Table 1. After filtering, 30,112 variants within 439 genes were identified with an average coverage of 47-fold, quality score of 175 and call rate of 0.97. As shown in Fig. 1, 37.4 % (11,261) of all called variants fell within coding regions with 22.4 % (6,745) being either missense (non-synonymous SNV) or nonsense (stop-gain and stop-loss) variants; 50.7 % (15,281) fell within regulatory 5’ or 3’ untranslated regions (UTR5 or UTR3, respectively). The majority of variants (59.8 %) were found within a single LLFS family (Additional file 1: Table S4) and (88.74 %, *n* = 26,723) were rare, occurring at <1 % in the general population, and 12,303 (46.04 %) were singletons (Additional file 1: Table S5).

**Table 1:** Filter Applied in LLFS sequence data

Among 464 Candidate genes, 448 genes with 48,918 variants sequenced	
Filter 1: ≥ 5x coverage	47 ± 36
Filter 2: ≥ 45 quality score	175 ± 53
Filter 3: ≥ 60 % call rate	97 % ± 7 %
Filter 4: Mendel Errors	MAF dependent
439 genes with 30,112 variants analyzed

#### Single variant analyses

**Fig. 2:**
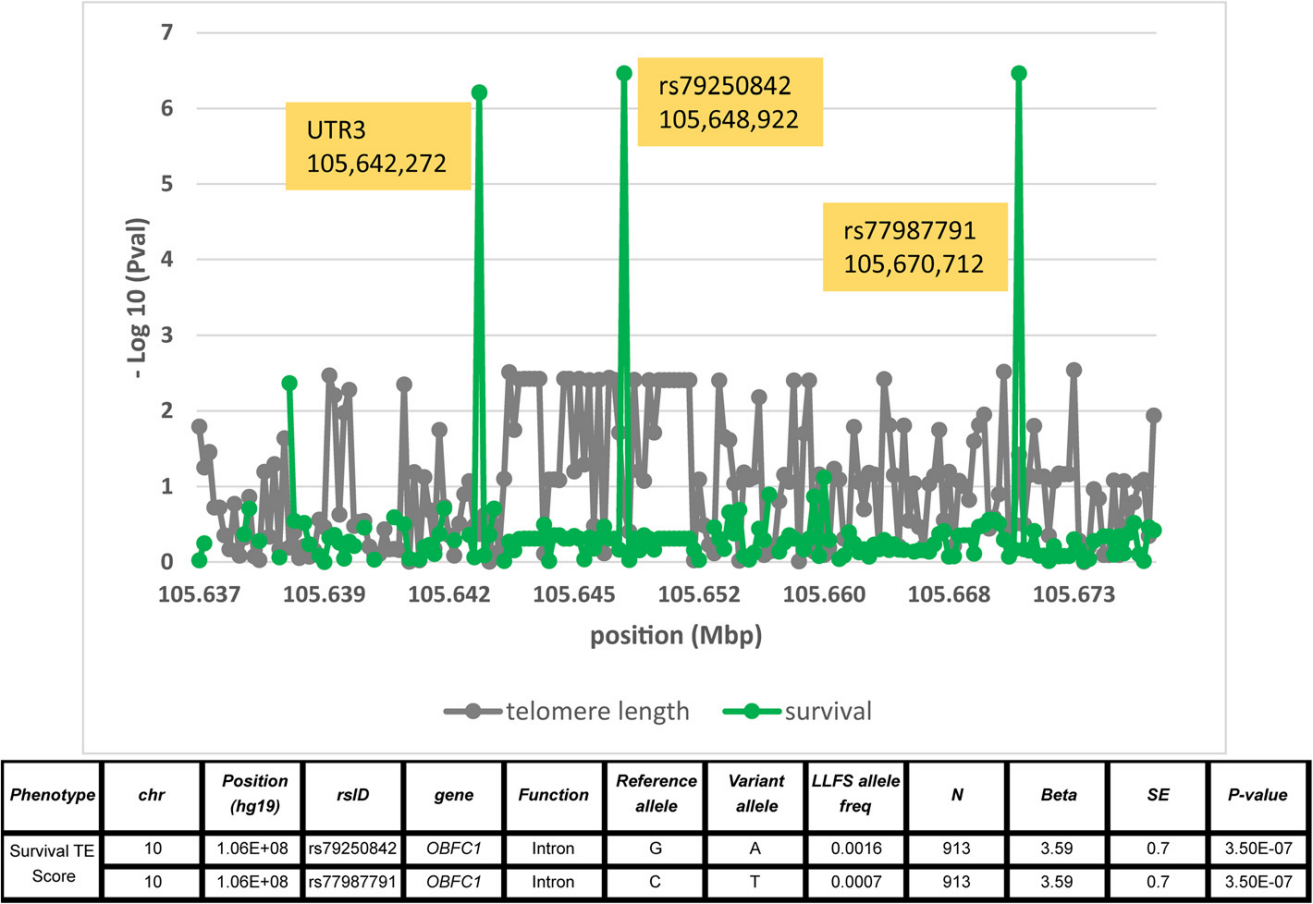
Variant-wise association results on chromosome 10 around *OBFC1* for telomere length phenotype (gray) and exceptional survival score (green) on chromosome 10 using both 1000 Genomes Project hybrid and candidate gene sequenced genotypes. Two additional rare variants from genome-wide array results showed significant association with survival exceptionality score. The association p-value of rs77987791 with telomere length is 0.038

Because there are correlations between the target phenotypes, the likelihood of a single variant having a functional effect on a given phenotype was Bonferroni corrected by dividing 0.05 by the total number of variants queried without accounting for the number of analyzed phenotypes. This resulted in p-values of 7.56x10^−6^ for survival and cancer and 1.47x10^−5^ for HDL, LDL and triglycerides. We first attempted to determine if any single variant identified by sequencing was significantly associated with survival. We identified a novel regulatory variant in the 3’ UTR of Oligonucleotide/Oligosaccharide-Binding Fold Containing 1, located on chromosome 10q24.33 (*OBFC1*; see Table 2) found in 13 individuals from six pedigrees (Additional file 1: Table S6), which was significantly associated (*p* = 6.18x10^−7^) with trait exceptionality for survival in long lived families. Intronic variants of *OBFC1* genotyped by Illumina array were also queried, and two rare variants (rs79250842 in 11 individuals from 5 pedigrees and rs77987791 in 5 individuals from 1 pedigree) were also found to have a significant association with survival (Fig. 2). *OBFC1* is involved in telomere maintenance and falls within a recently reported LLFS family-based association peak for telomere length on chromosome 10 as shown in Fig. 2 [16]. This observation is supported by the association of the same *OBFC1* intronic variant (rs77987791) with telomere length (*p* = 0.038). Interestingly, one long-lived pedigree (pedID 25609942) with 14 family members was clustered for *all three* rare variants in seven family members, which suggests that multiple rare variants within *OBFC1* might contribute to longer lifespan. The comparison of “survival” to *OBFC1* genotype is shown in Additional file 1: Figure S2A.

**Table 2:** Significant results of single variant testing for variants with more than 10 copies. Nine single variants with at least 10 allelic copies were identified within five phenotypes. There were 6,613 variants analyzed across 426 genes for survival and cancer and 3,389 variants analyzed across 408 genes for HDL, LDL and triglycerides

*Phenotype (TE Score)*	*chr*	*Position (hg19)*	*rsID*	*gene*	*Function*	*Ref*	*Variant*	*MAF*	*N*	*Beta*	*SE*	*P-value*
Survival	10	105642272		*OBFC1*	UTR3	C	G	0.002	810	3.58	0.71	6.2E-7
Cancer	15	99501295		*IGF1R*	UTR3	A	G	0.007	4210	0.58	0.13	3.7E-6
HDL Cholesterol	16	57005301	rs1532625	*CETP*	intron	T	C	0.38	3053	0.19	0.03	2.4E-13
	16	57015091	rs5880	*CETP*	nsyn-exon	C	G	0.047	4049	−0.32	0.06	1.2E-8
	16	57017319	rs1800777	*CETP*	nsyn-exon	A	G	0.029	3982	−0.40	0.07	8.1E-9
LDL Cholesterol	6	152679594	rs62426382	*SYNE1*	syn-exon	G	A	0.016	4035	0.45	0.09	1.6E-6
	19	45396144	rs11556505	*TOMM40*	syn-exon	T	C	0.10	4018	−0.19	0.04	1.5E-6
	19	45397229	rs1160983	*TOMM40*	syn-exon	A	G	0.02	2686	0.53	0.10	1.2E-7
Triglyceride	11	116703640	rs5128	*APOC3*	UTR3	G	C	0.09	4003	−0.21	0.04	1.2E-7

**Fig. 3:**
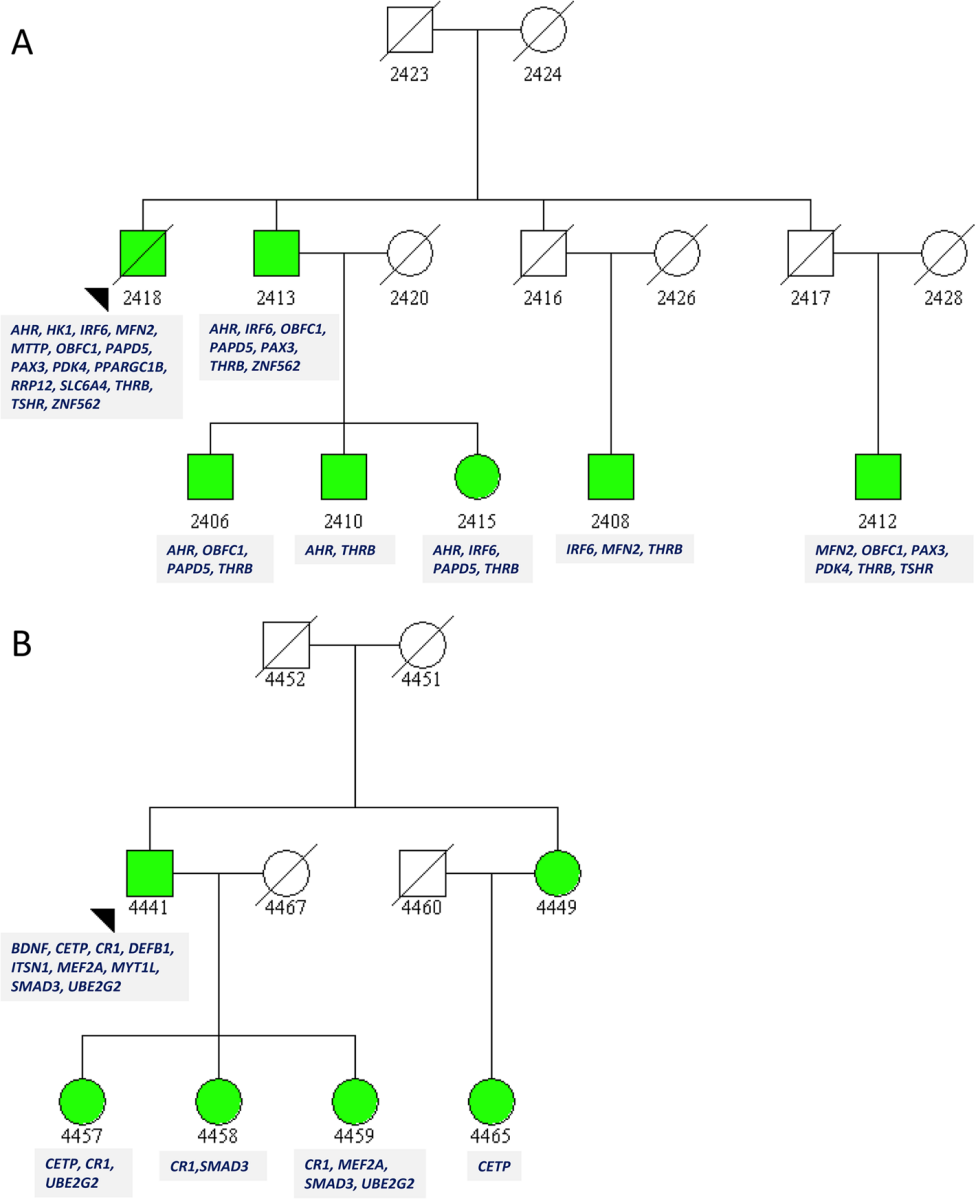
Twenty-four rare variants within twenty-four genes clustered in two long-lived LLFS families (**a**) pedID 25609942 and (**b**) pedID 38652533. Individual 2418 and 4441 lived to 110 and 101 years of age, respectively. The longevity-associated genes harboring rare variants within these individuals (and their offspring) are listed below their symbol

Linear mixed model based single variant testing is not a stable method for rare variants with <10 minor allele copies [28], which is strengthened by our pedigree-based structure. While the effect of any single variant may be slight, we found a significant association with survival for 23 different variants in 23 healthy aging candidate genes (Table 3). Among these 23 variants, only four have been described in dbSNP. Eight of these genes (in bold) have published reports linking them to longer lifespan [9, 29–34]. These variants clustered in two families with longer survival (Fig. 3), suggesting that the aggregation of multiple variants in these genes may have a larger effect on survival. One subject who survived to age 110 carried 15 of these variants, and another surviving to age 101 years carries 9 of these variants. None of the variants were carried by spousal controls.

**Table 3:** Significant results of single variant testing for variants with less than 10 copies. Twenty-three rare variants with less than 10 minor allele copies were identified for a single phenotype (longevity). These variants clustered in two families with high trait exceptionality scores for longevity. Genes in bold have a previously published relationship to longevity [9, 29–34]

*chr*	*Position (hg19)*	*rsID*	*gene*	*Function*	*Ref*	*Variant*	*Minor Allele Count*	*MAF*	*N*	*Beta*	*SE*	*P-value*
7	17385235		*AHR*	UTR3	A	G	5	0.0006	810	3.58	0.71	6.18E-07
11	27680107	rs8192466	***BDNF***	nsyn-exonic	A	G	8	0.0009	810	5.02	1.01	7.41E-07
16	57015065		***CETP***	intronic	T	C	3	0.0004	809	5.00	1.01	8.69E-07
1	207813049		*CR1*	UTR3	A	G	5	0.0006	810	5.02	1.01	7.41E-07
8	6735377		*DEFB1*	nsyn-exonic	T	C	1	0.0001	809	5.03	1.01	7.22E-07
10	71139772		*HK1*	nsyn-exonic	T	C	1	0.0001	794	5.04	1.01	6.53E-07
1	209959231		*IRF6*	UTR3	G	A	4	0.0005	810	3.58	0.71	6.18E-07
21	35195906		*ITSN1*	syn-exonic	A	C	1	0.0001	810	5.02	1.01	7.41E-07
15	100256347		*MEF2A*	UTR3	C	T	2	0.0002	810	5.02	1.01	7.41E-07
1	12073445		***MFN2***	UTR3	T	C	3	0.0003	810	5.07	1.01	6.10E-07
4	100544175		***MTTP***	UTR3	G	A	1	0.0001	810	5.07	1.01	6.10E-07
2	1794502		***MYT1L***	UTR3	T	C	1	0.0001	810	5.02	1.01	7.41E-07
16	50267300		***PAPD5***	UTR3	T	C	4	0.0005	810	3.58	0.71	6.18E-07
2	223158616		*PAX3*	UTR3	T	C	3	0.0003	810	3.58	0.71	6.18E-07
7	95214257		*PDK4*	UTR3	G	A	2	0.0002	810	5.07	1.01	6.10E-07
5	149206403	rs374853976	*PPARGC1B*	syn-exonic	A	G	1	0.0001	714	5.07	1.00	4.72E-07
10	99130549		*RRP12*	nsyn-exonic	T	C	1	0.0001	780	5.02	1.01	7.66E-07
17	28525015	rs199875985	***SLC6A4***	UTR3	G	A	1	0.0001	810	5.07	1.01	6.10E-07
15	67479818	rs144245324	*SMAD3*	syn-exonic	T	C	3	0.0004	767	5.02	1.02	9.22E-07
3	24163883		*THRB*	UTR3	T	C	7	0.0008	810	3.58	0.71	6.18E-07
14	81610971		***TSHR***	UTR3	A	G	2	0.0002	810	5.07	1.01	6.10E-07
21	46189291		*UBE2G2*	UTR3	T	C	3	0.0004	810	5.02	1.01	7.41E-07
19	9770038		*ZNF562*	intronic	T	G	2	0.0002	810	3.58	0.71	6.18E-07

Among the 439 candidate genes, 160 are well-known human longevity related genes based on LongevityMap (http://genomics.senescence.info/longevity/) [35] and A Catalog of Published Genome-Wide Association Studies (http:/www.genome.gov/gwastudies/). When considering survival and any single variant with a p-value of <0.05 and *more than* 10 allelic copies in the LLFS cohort, we replicated 76 variants within these 160 genes (Additional file 1: Table S7), which supports the validity of our analysis model. Four variants within *FOXO3*, a well-known longevity related gene [36–39], were replicated for an association (*p* < 0.05) with survival.

We found associations with phenotypes other than survival. One candidate gene, *IGF1R* (Insulin-Like Growth Factor 1 Receptor, located on chromosome 15q26.3) is well known to be associated with cell growth and various cancers [40]. As shown in Table 1, we noted one new cancer associated rare UTR3 variant (chr15:99501295) in this gene.

In addition, four genes (*CETP, APOC3, SYNE1 and TOMM40*) were associated with blood lipid levels. The cholesterol ester transfer protein, *CETP* (located on chromosome 16q21), is essential for metabolism of plasma lipoproteins [41]. Three known HDL cholesterol related variants within *CETP*, one common intronic variant (rs1532625) [42] and two rare exonic variants (rs5880, rs1800777) [43], were replicated in our study. *APOC3* (Apolipoprotein C3, located on chromosome 11q23.3) is critical for triglyceride metabolism and a potential therapeutic target for metabolic syndrome [44]. One known common triglyceride related variant (rs5128) [45] in the UTR3 region of *APOC3* was replicated. LDL cholesterol was associated with one synonymous variant in *SYNE1* (Spectrin Repeat Containing, Nuclear Envelope 1, on chromosome 6q25.2) and two synonymous variants in *TOMM40* (Translocase Of Outer Mitochondrial Membrane 40 Homolog, on chromosome 19q13.32). *TOMM40* has been identified in genome-wide screens for dyslipidemia and carotid artery disease [46, 47]. More importantly, *TOMM40* rs10524523 polymorphism in combination with *APOE* alleles significantly influences late-onset Alzheimer’s disease and longevity [48]. Comparisons for all variants listed in Table 2 between their associated phenotype and the variant genotype are shown in Additional file 1: Figure S2A-I.

#### Analyses of multiple variants across genes

To better characterize genes associated with our phenotypes due to multiple, pedigree-specific rare variants rather than more common single variants, we aggregated rare functional variants (exonic, UTR3, UTR5, splicing and non-coding RNA) within the same gene using the PWST [25] and famSKAT [26]. For association results, Bonferroni correction of 0.05 divided by the total number of genes resulted in a *p* < 1.14x10^−4^ for significance and *p* < 1.0x10^−3^ would merely be suggestive. With respect to survival, none of the candidate genes passed this significance threshold. When comparing our association results for survival to published longevity related genes, 20 genes were replicated with p-value <0.05 (Additional file 1: Table S8).

We next performed PWSK and famSKAT analyses on our candidate genes to see if any gene harbored multiple putatively functional variants associated with the individual Healthy Aging Index (HAI) and those results are listed in Table 4. *GSK3B* (glycogen synthase kinase 3 beta, on chromosome 3q13.33) achieved significance by PWSK. *GSK3B*, a serine-threonine kinase, has been associated with Alzheimer’s disease [49, 50] and may regulate human aging via negative regulation of glucose homeostasis and Wnt signaling. In addition, *NOTCH1* was significantly associated with diastolic blood pressure and *TP53* with HDL (Table 4). *NOTCH1* (chromosome 9q34.3) is involved in a variety of developmental processes by controlling cell fate. In mice, *NOTCH1* signaling is also required for vascular development [51]. *TP53* (tumor protein P53, located on chromosome 17p13.1), is a well known tumor suppressor that regulates cell cycle and DNA repair. An inverse correlation of HDL cholesterol with cancer risk [52] might be explained by the association of *TP53* with HDL cholesterol.

**Table 4:** Collapsed rare variant analysis of candidate genes associated with HAI. These genes were found to have a *p*-value <1.0x10^−3^ by at least one burden-testing algorithm

*Phenotype*	*Gene*	*RefSeq*	*Total Exons*	*Analyzed Exons*	*Indiv Seq (#)*	*Variants called (#)*	*Ped (#)*	*P-value (PWST)*	*P-value (Skat)*
Healthy Aging Index (Mortality Weighted)	*GSK3B*	NM_002093	12	8	3217	77	121	1.00E-4	2.08E-4
Diastolic Blood Pressure	*NOTCH1*	NM_017617	34	3	4112	55	78	1.00E-4	5.37E-3
HDL Cholesterol	*TP53*	NM_000546	11	6	4073	21	86	1.00E-4	8.05E-3

These results are likely an underestimate of significant or suggestive variants. Unfortunately, non-uniform hybridization to target loci due to local sequence context and the need for stringent filtering to reduce false positives resulted in gaps within the covered coding sequence of most genes, which could affect results due to false negatives. This is why the well-known longevity gene, *ApoE* [53], was not included in our analyses.

## Discussion

The mechanisms driving healthy human aging are still mostly unclear. There is much debate on the evolutionary versus adaptive mechanisms of aging, such as programmed longevity, hormonal regulation of aging by insulin/IGF-1 signaling, programmed decline of the immune system, wear and tear theory, rate of oxygen basal metabolism, cross-linking theory and free radicals theory. These mechanisms are not inherently mutually exclusive and it is likely that aging is dictated by combinations of multiple mechanisms. Regardless, Kirkwood and Melev stated in a recent review that “it is obvious…that duration of life is dependent upon genotype” [54].

To take advantage of the unique family structure of the LLFS cohort, we have performed candidate gene resequencing to identify familial sequence variation that could explain multiple exceptional phenotypes contributing to long lifespan and healthy aging. Our results are limited by gaps in sequencing coverage of some genes due to non-uniform hybridization of baits across the entire cohort, which may mean an underestimate in the number of familial variants in these genes. This strategy was previously used to identify rare familial variants in *VEGFC* (Vascular Endothelial Growth Factor C) by using linkage information in families with high LOD scores to inform targeted resequencing for rare variants. This experiment identified a familial variant in *VEGFC* that explained 23.8 % of phenotypic variance within a pedigree, but the same variant only described 0.1 % of the phenotypic variance in unrelated individuals [55]. The same strategy was employed in the Insulin Resistance Atherosclerosis Family Study to identify a rare variant within the *ADIPOQ* (Adiponectin, C1Q And Collagen Domain Containing) gene describing 63 % of the variance of plasma adiponectin levels, which are critical for glucose homeostasis [56]. Our results validate multiple SNPs and genes (*OBFC1, CTEP, ZNF562*) found associated with longevity and healthy survival. Using a sequencing-based approach to identify rare variants within pedigrees associated with exceptional phenotypes. Of note, we identified one rare, novel variant in *OBFC1* as well as two intronic variants from LLFS array data associated with survival. *OBFC1* is involved in telomere maintenance; its role in promoting exceptionally healthy aging is intriguing given that this gene resides squarely within a family-based association peak for telomere length recently reported from the same LLFS families [16]. The idea that many longevity-associated variants can presumably work in synergy with an additive beneficial effect is supported by the observations from three LLFS families. One exceptionally long-lived LLFS family carries all three *OBFC1* variants, while 23 *additional* beneficial rare variants are clustered in two other exceptionally long-lived LLFS families.

Because effect sizes from single rare variants are typically small, collapsing rare variants across larger loci (e.g. genes or pathways) can aggregate variants and highlight the importance of a larger genetic locus [25]. By analyzing our sequencing results in this fashion, we identified a more diverse set of genes associated (or suggestive of association) with additional healthy aging phenotypes. *GSK3B*, which is a kinase for over forty different proteins and plays key roles in numerous intracellular signaling pathways (cellular proliferation, migration, inflammation and immune response, glucose regulation, and apoptosis) [57] was identified in this analysis and may play an important role in healthy aging. This gene has been associated with a number of age-related diseases such as type II diabetes, Alzheimer’s disease, inflammatory disorders, cancer and bipolar disorder [58].

Another healthy aging candidate gene, *CETP*, is a well-known regulator of HDL and has been associated with healthy aging in Ashkenazi Jewish [59, 60] as well as Alzheimer’s disease [61]. The negative association of several variants with HDL, LDL and triglycerides in our study indicates that harmful genetic variants still exist in the long-lived subjects. This paradoxical phenomenon might be due to antagonistic effects on the development of other age-related disorders, gene-age, gene-gene, or gene-environment interactions [62]. To understand these intersections, further investigation of age and environment specific effects of these lipid regulatory genes on multiple aging phenotypes is needed.

## Conclusions

Under the Rare Variant/Complex Phenotype hypothesis, multiple rare variants have an aggregate effect on complex phenotypes or diseases, but identifying the genes and the relative contributions of the various sequence changes is difficult. By leveraging the pedigree structure and cohort size of the LLFS study, we demonstrate the utility of sequencing within pedigrees to identify inherited genetic variation influencing specific parameters of healthy aging. These data are limited by representing primarily genes that were already known to be associated with the various phenotypes being investigated. A larger survey of the genome in the LLFS cohort, especially the linkage peaks for multiple healthy aging phenotypes, will facilitate discovery of new genes and putative mechanisms for the genetic regulation of the complexities of aging.

### Ethics approval and consent to participate

Each participant provided written, informed consent prior to inclusion in this study. This study is in compliance with the Helsinki Declaration and the results reported herein have been approved by the National Institute of Aging, the Human Research Protection Office of the coordinating center at Washington University under IRB#201106316, the University of Pittsburgh Institutional Review Board, the Boston University Office of the Institutional Review Board, the Columbia University Institutional Review Board and the Regional Scientific Ethical Committees for Southern Denmark.

### Consent for publication

Not applicable.

### Availability of data

GWA and sequencing information for the LLFS participants is available in dbGaP using accession number phs000397.v1.p1.

## Additional file

Additional file 1: Supplementary Materials. (PDF 1024 kb)

## Abbreviations

LLFS: Long Life Family Study
HDL: high density lipoprotein
LDL: low density lipoprotein
BMI: body mass index
TIA: transient ischemic attack
MMSE: mini mental state examination
UTR: untranslated region
FLoSS: family longevity selection score
HAI: healthy aging index
GWAS: genome wide association study
PCs: principal components
MAF: minor allele frequency
HgbA_1C_: glycated hemoglobin
UWSS: un-weighted sum statistic
WSS: weighted sum statistic
PWST: P-value weighted sum test
famSKAT: family-based serial kernel association test
