# Supplementary material for: Candidate gene resequencing to identify rare, pedigree-specific variants influencing healthy aging phenotypes in the long life family study
Source: BMC Geriatr. 2016 Apr 9;16:80. doi: 10.1186/s12877-016-0253-y (PMC4826550; doi:10.1186/s12877-016-0253-y)
Supplement: Supplementary file 1 — Supplementary Materials. (PDF 1024 kb) [file 12877_2016_253_MOESM1_ESM.pdf]

SUPPLEMENTARY INFORMATION FOR:

Candidate gene resequencing to identify rare, pedigree-specific variants influencing healthy aging phenotypes in the Long Life Family Study

Todd E. Druley (druley_t@kids.wustl.edu)[1,2], Lihua Wang (juwang@dsgmail.wustl.edu)[1,3], Shiow J. Lin (rosa@dsgmail.wustl.edu)[1,3], Joseph H. Lee (jhl2@columbia.edu)[4], Qunyuan Zhang (qunyuan@dsgmail.wustl.edu)[1,3], E. Warwick Daw (warwick@wustl.edu)[1,3], Haley J. Abel (habel@dsg.wustl.edu)[1,3], Sara E. Chasnoff (chasnoff_s@kids.wustl.edu)[1,2], Enrique I. Ramos (eiramos@wustl.edu)[1,2], Benjamin T. Levinson (benjaminlevinson2010@u.northwestern.edu)[1,2], Bharat Thyagarajan (thya0003@umn.edu)[5], Anne B. Newman (newmana@edc.pitt.edu)[6], Kaare Christensen (kchristensen@health.sdu.dk)[7], Richard Mayeux (rpm2@cumc.columbia.edu)[8], Michael A. Province (mprovince@wustl.edu)[1,3]

[1] Center for Genome Sciences and Systems Biology, Washington University School of Medicine, USA.

[2] Department of Pediatrics, Washington University School of Medicine, USA.

[3] Division of Statistical Genomics, Department of Genetics, Washington University School of Medicine, USA.

[4] Sergievsky Center, College of Physicians and Surgeons, Columbia University New York, NY, USA ; Taub Institute, College of Physicians and Surgeons, Columbia University New York, NY, USA ; Department of Epidemiology, School of Public Health, Columbia University New York, NY, USA.

[5] Department of Laboratory Medicine and Pathology, University of Minnesota, Minneapolis, MN, USA.

[6] Department of Epidemiology, University of Pittsburgh Graduate School of Public Health Pittsburgh, PA, USA.

[7] The Danish Aging Research Center, Epidemiology, University of Southern Denmark Odense, Denmark.

[8] Gertrude H. Sergievsky Center and the Taub Institute for Research on Alzheimer's Disease and the Aging Brain, Columbia University, New York City, NY, USA.

Supplementary Table 1. Characteristics (Mean ± standard deviation) of Subjects in LLFS Study by generation. #LLFS proband generation including probands and probands' relatives. **corrected for medication use as published by Wu J *et al.* Am J Hypertens **18**(7): 935-942. Referenced under *Methods: Subjects*.

**Phenotypes**	Proband Generation			Offspring Generation			Controls		
	Family (#)	Subject (#)	Mean ± STD	Family (#)	Subject (#)	Mean ± STD	Family (#)	Subject (#)	Mean ± STD
Age, yrs	549	1240	90.26 ± 6.50	509	2119	60.63 ± 8.21	313	863	65.11 ± 12
HbA1c (%)	513	1063	5.63 ± 0.36	486	1898	5.46 ± 0.32	289	760	5.5 ± 0.33
Telomere Length	500	1129	5160.91 ± 383.90	480	1960	5412.17 ± 476.81	296	812	5300.51 ± 454.2
Healthy Aging Index	492	922	5.76 ± 1.76	467	1609	2.61 ± 1.68	272	694	3.2 ± 1.91
Healthy Aging Index (Mortality Weighted)	492	922	5.57 ± 1.88	467	1609	1.96 ± 1.52	272	694	2.58 ± 1.90
Body Mass Index	537	1158	26.01 ± 4.20	503	2026	27.66 ± 5.27	310	847	27.44 ± 4.61
Systolic Blood Pressure**	544	1215	153.01 ± 27.76	506	2052	134.40 ± 22.86	309	849	140.72 ± 25.13
Diastolic Blood Pressure**	544	1215	83.73 ± 13.37	506	2052	83.41 ± 12.39	309	849	85.02 ± 12.90
Pulse Pressure**	544	1215	69.28 ± 21.15	506	2052	50.99 ± 15.28	309	849	55.70 ± 17.47
Cognitive Function	535	1146	25.18 ± 4.32	495	1788	28.91 ± 2.28	293	765	28.5 ± 2.27
HDL Cholesterol (mg/dl)**	541	1204	55.12 ± 16.26	498	2034	60.09 ± 18.06	306	839	58.53 ± 16.88
LDL Cholesterol (mg/dl)**	541	1196	118.64 ± 35.75	498	2013	131.94 ± 34.99	306	830	132.73 ± 34.98
Triglyceride (mg/dl)**	541	1204	118.38 ± 65.70	498	2034	123.41 ± 84.73	306	839	124.65 ±83.33
Total Cholesterol (mg/dl)**	541	1204	199.00 ± 43.40	498	2034	216.79 ± 40.01	306	839	216.5 ± 40.66
Glucose (mg/dl)	543	1212	95.91 ± 21.55	502	2068	94.43 ± 20.29	306	846	96.99 ± 20.98
Insulin (mU/L)	542	1200	8.32 ± 8.76	501	2049	9.11 ± 7.70	303	842	9.1 ± 6.98
Onset age of Stroke*	549	188	82 ± 13.15	509	54	57.68 ± 11.15	313	44	62.86 ± 15.53
Onset age of Heart Disease*	549	214	76.76 ± 12.91	509	80	57.68 ± 11.15	313	65	63.2 ± 11.86
Onset age of Cancer*	549	257	74.64 ± 14.62	509	191	53.47 ± 13.09	313	90	62.04 ± 11.93
Onset age of Chronic Heart Failure*	549	138	86.02 ± 10.86	509	21	62.67 ± 9.97	313	17	69.53 ± 13.73
Onset age of Diabetes*	543	127	77.42 ± 12.79	503	134	55.80 ± 13.85	306	74	61.73 ± 14.13
Onset age of Death*#	498	431	97.56 ± 3.70						

Supplementary Table 2. Characteristics (Mean ± standard deviation) of Subjects in LLFS Study by gender. #LLFS proband generation including probands and probands' relatives**.** **corrected for medication use as published by Wu J *et al.* Am J Hypertens **18**(7): 935-942. Referenced under *Methods: Subjects*.

Phenotypes	Men			Women		
	Family (#)	Subject (#)	Mean ± STD	Family (#)	Subject (#)	Mean ± STD
Age, yrs	507	1899	70.56 ± 15.11	527	2318	70.05 ± 16.09
HbA1c (%)	472	1646	5.52 ± 0.35	511	2075	5.52 ± 0.33
Telomere Length	474	1749	5277.32 ± 447.52	497	2150	5347.67 ± 467.36
Healthy Aging Index	465	1426	3.64 ± 2.22	481	1791	3.64 ± 2.22
Healthy Aging Index (Mortality Weighted)	465	1426	3.24 ± 2.31	481	1791	3.03 ± 2.32
Body Mass Index	499	1822	27.58 ± 4.13	518	2209	26.78 ± 5.43
Systolic Blood Pressure**	503	1855	140.41 ± 23.62	522	2261	141.84 ± 27.99
Diastolic Blood Pressure**	503	1855	85.58 ± 12.53	522	2261	82.41 ± 12.85
Pulse Pressure**	503	1855	54.84 ± 17.4	522	2261	59.43 ± 20.74
Cognitive Function	492	1640	27.59 ± 3.25	513	2059	27.73 ± 3.67
HDL Cholesterol (mg/dl)**	498	1839	51.96 ± 15.27	520	2238	63.51 ± 17.38
LDL Cholesterol (mg/dl)**	498	1811	124.78 ± 34.16	520	2228	130.91 ± 36.76
Triglyceride (mg/dl)**	498	1839	127.48 ± 90.79	520	2238	117.82 ± 68.15
Total Cholesterol (mg/dl)**	498	1839	202.55 ± 39.60	520	2238	218.82 ± 42.40
Glucose (mg/dl)	502	1859	97.72 ± 21.92	522	2267	93.47 ± 19.69
Insulin (mU/L)	501	1845	9.15 ± 8.41	519	2246	8.65 ± 7.43
Onset age of Stroke*	506	120 (1894)	71.25 ± 15.59	527	166 (2319)	75.78 ± 18.71
Onset age of Heart Disease*	506	215 (1890)	68.33 ± 13.11	527	144 (2318)	72.63 ± 16.90
Onset age of Cancer*	506	248 (1894)	68.67 ± 14.42	527	290 (2321)	61.89 ± 17.88
Onset age of Chronic Heart Failure*	506	82 (1892)	77.59 ± 14.46	527	94 (2316)	85.18 ± 12.45
Onset age of Diabetes*	502	180 (1868)	65.06 ± 16.00	524	155 (2279)	65.59 ± 17.38
Onset age of Death*#	334	224 (425)	95.83 ± 3.28	292	207 (385)	99.43 ± 3.19

Supplementary Table 3. Candidate genes sequenced by custom hybridization capture. Referenced under *Methods: Pooled sequencing and indexed custom library preparation*.

A2M	C4orf34	DEFB1	FOS	IDH3G	MDH2	PAX2	RAD21	TAX1BP
ABCA1	C9orf11	DGAT1	FOXA1	IGF1	MEF2A	PAX 3	RAD51L1	TBL1XR1
ACADM	C9orf3	DGAT2	FOXA2	IGF1R	MEF2C	PAX 7	RAD51L3	TBXAS1
ACADVL	C9orf94	DKC1	FOXA3	IGF2	MEF2D	PCK2	RAE1	TEK
ACCN1	CADPS2	DKK1	FOXC1	IGSF4	MEOX2	PCNA	RRP12	TERC
ACE	CALHM1	DLG7	FOXC2	IL10	METAP1	PCNXL2	RUNDC1	TERF1
ACOT1	CAMTA1	DLST	FOXK1	IL12A	MFN2	PDHB	RXRA	TERF2
ACOX1	CARD14	DMRT1	FOXO1	IL1A	MINPP1	PDK4	S100B	TERT
ADARB1	CAT	DNMT3a	FOXO3	IL1B	MIR181A2	PEO1	SDCBP2	TF
ADARB2	CD36	DNMT3b	FOXO4	IL6	MKL1	PFKFB3	SDHC	TFAM
AdipoQ	CDK6	DOCK2	FOXO6	INS	MMP10	PFTK1	SEMA6A	THRB
ADRB2	CDKN2A	DOCK8	FST	INSR	MMP7	PGAM1	SERINC3	TMEM2
AHR	CEACAM16	DPT	FTO	IRF6	MNAT1	PGAM2	SH2D4A	TMTC3
AIF1	CEBPB	DRD2	FUS	IRS1	MOCS1	PGK1	SH3GLB1	TNF
AKT1	CETP	DSG1	GAB2	IRS2	MSRA	PGPEP1	SH3RF2	TNFRSF11
AKT2	CGNL1	DTNBP1	GATA4	ITGA1	MTHFR	PGR	SHC1	TNFSF8
AKT3	CHRM2	DYNLL1	GBA3	ITSN1	MTOR	PHYHIP	SIRT1	TOMM40
ALAD	CHST11	DYNLL2	GCLM	JUN	MTTP	PICALM	SIRT2	TORC1
AMPK	CIDEA	DZIP3	GH1	KAT2A	MYBBP1A	PIG38	SIRT3	TORC2
ANGEL1	CLK2	EBF3	GHR	KCTD1	MYC	PIK3C3	SIRT4	TOX
APBB2	CLPTM1L	EEF1A1	GIP	KIAA0020	MYF5	PIK3CB	SIRT5	TP53
APOA4	CLU	EFEMP1	GK	KIAA0174	MYF6	PIK3IPI	SIRT6	TP63
APOC1	CLYBL	EFTUD1	GPC6	KIAA1377	MYO9B	PITPNM3	SIRT7	TRIM25
APOC-3	CNTN5	EIF4E3	GRIN2B	KIF13B	MYOD1	PLAU	SLC25A21	TRRAP
APOE	COMMD4	EIF5	GRN	KL	MYT1L	PLEKHA1	SLC4A4	TSC22D1
APOE-2	COMP	ELAVL1	GSK3B	KLRF1	NAV2	PLEKHA7	SLC6A3	TSHB
ARHGAP1	COMT	ELF2	GSTM1	LARGE	NBEA	PLEKHG3	SLC6A4	TSHR
ARNTL	COQ7	ELL2	GSTP1	LASS3	NCOR1	PLK3	SLC6A7	TTC6
ATG2B	CPT1A	ENO1	GSTT1	LBH	NDUFA11	PMP2	SMAD1	TUBB
ATM	CPT1B	EP300	H2AFX	LDHB	NFKB1	POLG	SMAD2	TUSC3
ATP2B4	CPT2	EPB41L4B	H6PD	LDLR	NFKBIE	PON1	SMAD3	UBE2A
ATP5O	CR1	ERCC2	HADHA	LEF1	NFKBIZ	POT1	SMAD5	UBE2G2
ATR	CREB1	ERCC8	HADHB	LIPC	NOTCH1	POU1F1	SMARCE1	UBE2H
AVPI1	CREBBP	ERGIC1	HDDC2	LLGL1	NPM1	POU2F1	SNF1LK	UCGC
BACE1	CREBZF	ESR1	HELLS	LMNA	NR0B1	POU5F1	SOCS2	UCP2
BAT5	CSF1R	ESRRA	HK1	LMX1B	NR1D1	PPARG c1a	SOD1	ULK1
BCHE	CSMD3	ESRRG	HK2	LOC285429	NR1H3	PPARD	SOD2	VASH1
BDNF	CSNK1G2	EXOSC6	HK3	LOC387763	NR1H4	PPARG	SORCS1	VPS13D
BHLHE40	CST3	FABP1	HLA-DRB1	LOC654121	NR2F1	PPARGC1A	SORCS2	VPS72
BIN1	CTBP1	FABP2	HNF1A	LOC728734	NR2F2	PPARGC1B	SORL1	WDR72
BRCA1	CTBP2	FABP3	HNF1B	LRP1	NR3C1	PPP2R2B	SOX18	WEE1
BRE	CTDSP2	FABP4	HRAS	LRP1B	NR4A2	PPTC7	SOX9	WNT 5B
BTG3	CTNNA1	FABP5	HS3ST3B1	LRPPRC	NRF1	PRDM16	SREBF1	WRN
BTNL2	CTNNA2	FABP7	HSF1	LRRN6C	NT5DC1	PREX1	SREBF2	WWC1
BUB1B	CTNNA3	FAHD1	HSPA9	LYST	OBFC1	PRNP	SSPN	WWOX
BUB3	CTNND2	FAS	HSPB1	LZTR1	OGDH	PROM1	STAT1	XDH
C12orf31	CYP1A1	FBX032	HSPC159	MAFA	ONECUT1	PROP1	STUB1	XKR6
C12orf72	CYP3A5	FGFR1	hTERC	MAFF	OR56A1	PROX1	SUMF1	XRCC5
C1orf151	DAB2	FHIT	hTERT	MAPK8	PAK1	PTEN	SYNE1	ZAK
C2	DCPS	FLJ20272	HTT	MAPK9	PAPD5	PTK2	SYT13	ZBTB20
C20orf79	DDB1	FLJ31951	IDE	MAPT	PAPPA2	PYGO2	TADA2A	ZNF562
C21orf57	DDIT3	FLJ33708	IDH3A	MB	PARK2	RAB9A	TAPBPL	ZWINT

Supplementary Figure 1. Q-Q Plots

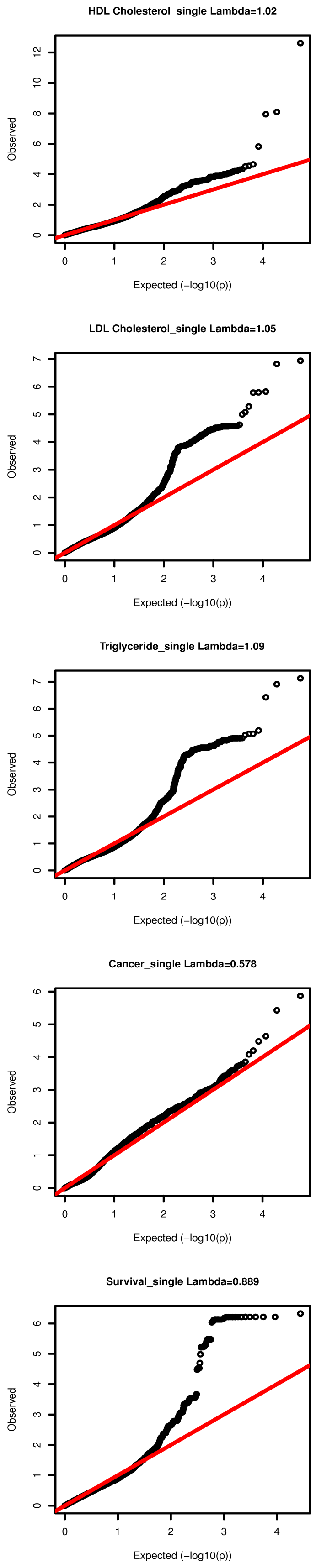

Supplementary Table 4. The distribution of variants within families in the sequence data. Referenced under *Results: Sample characteristics: Sequencing results*.

Category	Count	Percent
Singletons	12,303	40.86
Variants in 1 Family	17,995	59.76
Variants in 2 Familes	2,997	9.95
Variants in 3 Families	1,264	4.20
Variants in 4 Families	803	2.67
Variants in 5 Families	477	1.58
Variants in 6 Families	397	1.32
Variants in 7 Families	292	0.97
Variants in 8 Families	236	0.78
Variants in 9 Families	211	0.70
Variant in more than 10 Families	5,440	18.07

Supplementary Table 5. The distribution of allele frequency in the sequence data. Referenced under *Results: Sample characteristics: Sequencing results*.

MAF (%)	Count (Percent): Longevity Family	Count (Percent): Control Group
0 - 0.5	26035 (86.46)	26021 (86.41)
>0.5 – 1.0	696 (2.31)	706 (2.34)
>1.0 – 5.0	1268 (4.21)	1290 (4.28)
>5.0 – 10.0	481 (1.60)	472 (1.57)
>10.0 – 30.0	999 (3.32)	999 (3.32)
>30.0 – 50.0	633 (2.10)	624 (2.07)
Total	30112 (100)	30112 (100)

Supplementary Table 6. Pedigrees and individuals with the novel *OBFC1.* Only two individuals – brothers – with this variant are deceased (2413 and 2418). Referenced under *Results: Sample characteristics: Single variant analyses*.

PEDID	fuscore_adj1	subject	AGE	GENDER	DADsubj	MOMsubj
25609942	2.08325539	2413	97	male	2423	2424
25609942	5.07095304	2418	110	male	2423	2424
25609942	NA	2406	57	male	2413	2420
25609942	NA	2412	65	male	2417	2428
30706733	NA	8049	62	female	8047	8054
30706733	NA	8050	61	male	8046	8048
30706733	NA	8054	89	female	8044	8045
25826322	NA	11291	75	female	0	0
25826322	NA	11293	51	female	11283	11291
25562597	NA	11103	61	male	10081	11101
25562597	NA	30289	72	male	10081	11101
2162	NA	12783	72	female	0	0
21658323	NA	30673	58	female	30666	30672
6 pedigrees			13 people			

Supplementary Fig 1. Boxplots of associated traits vs candidate genotypes. Referenced under *Results: Sample characteristics: Single variant analyses*.

X axis 0: variant/variant genotype

X axis 1: ref/variant genotype

X axis 2: ref/ref genotype

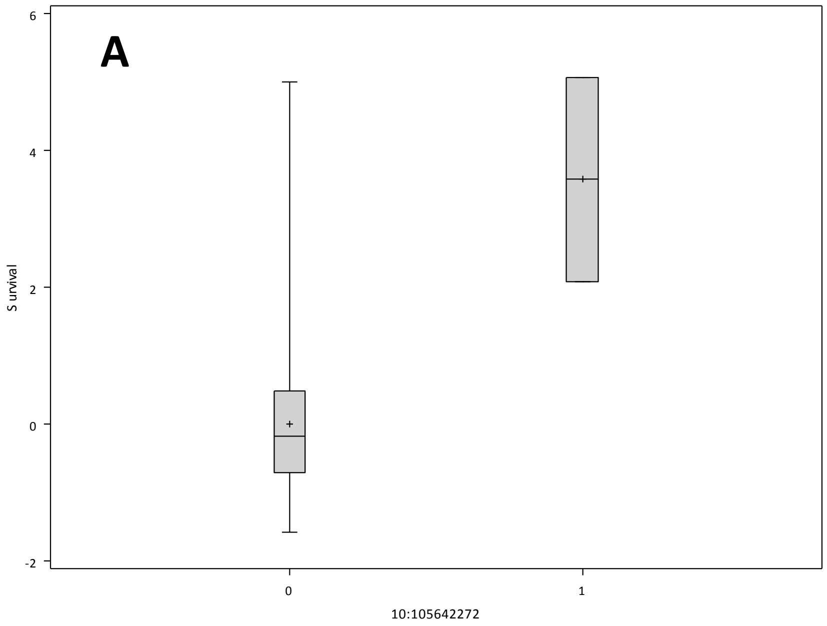

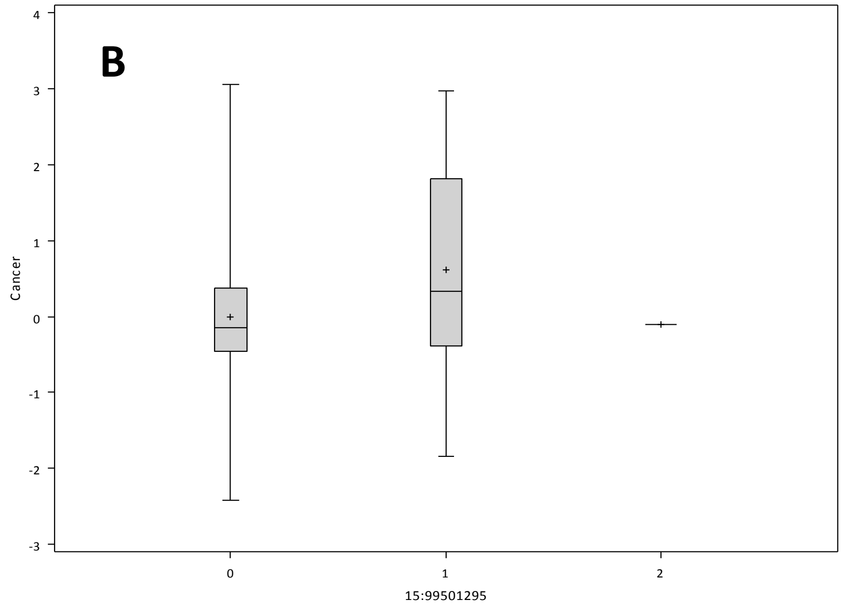

Supplementary Fig 1. Boxplots of traits vs candidate dosage of ref allele (Continued)

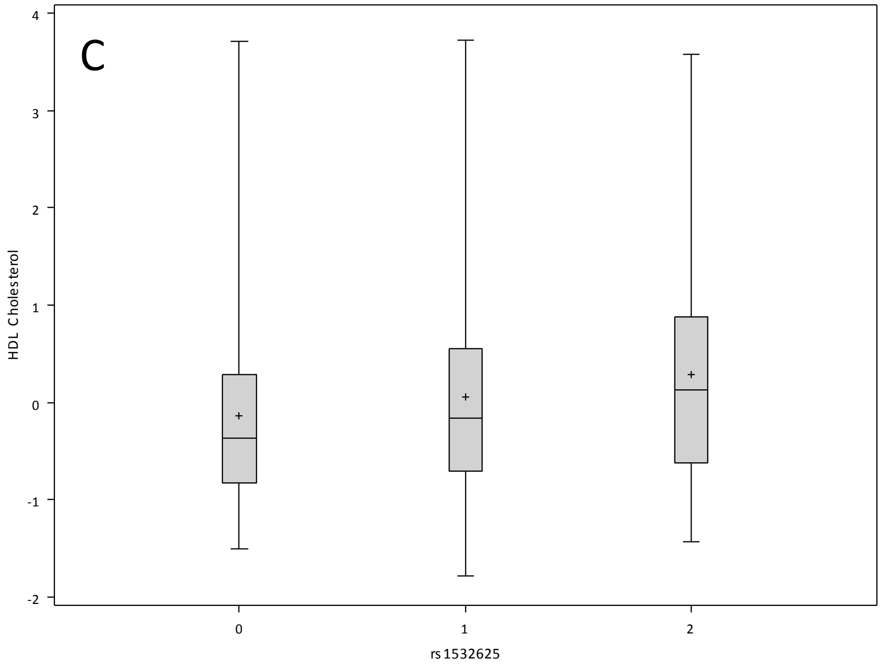

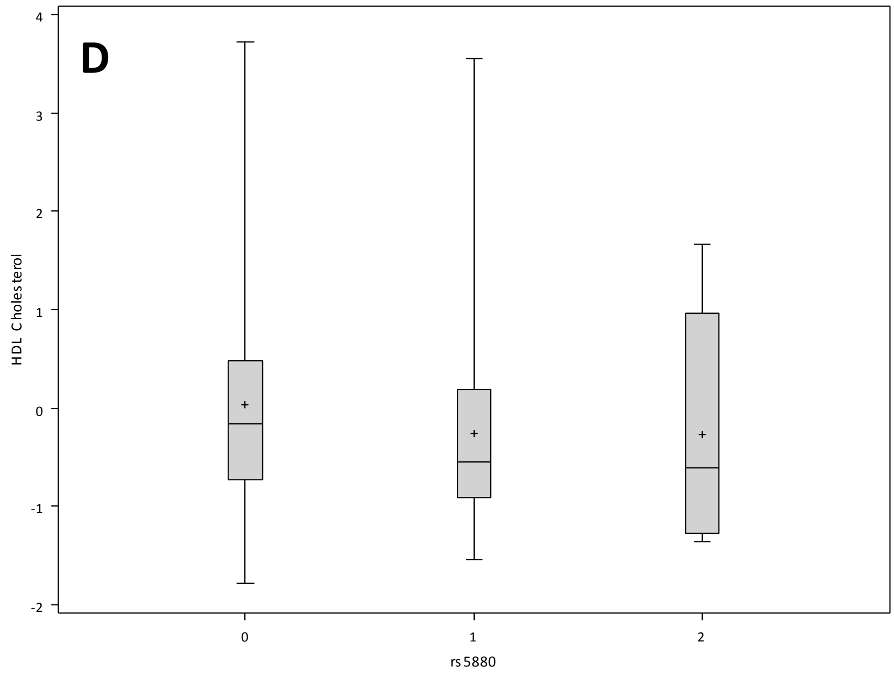

Supplementary Fig 1. Boxplots of traits vs candidate dosage of ref allele (Continued)

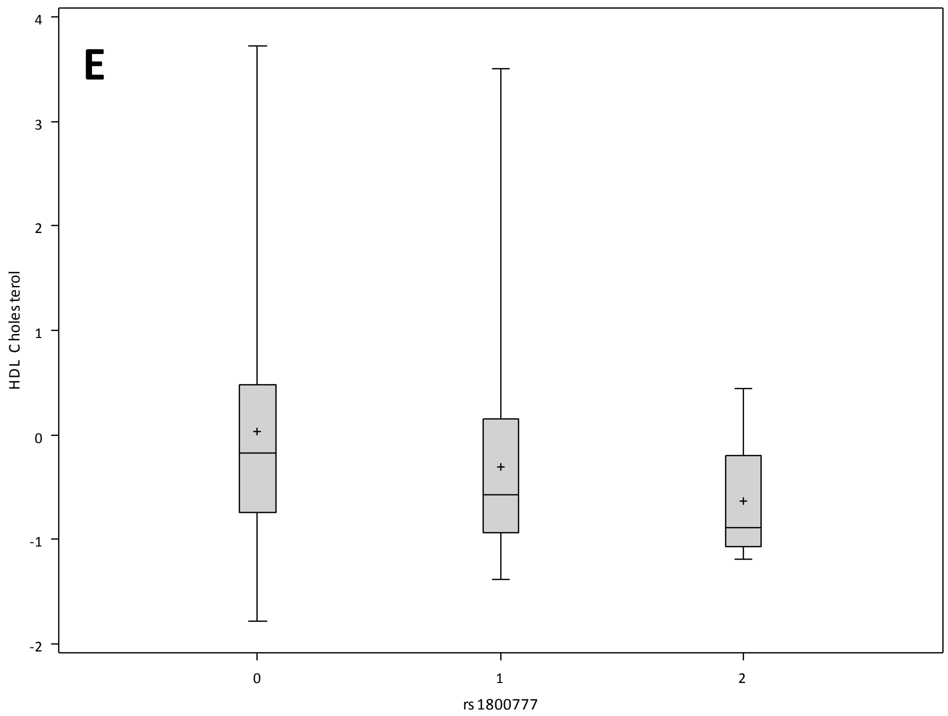

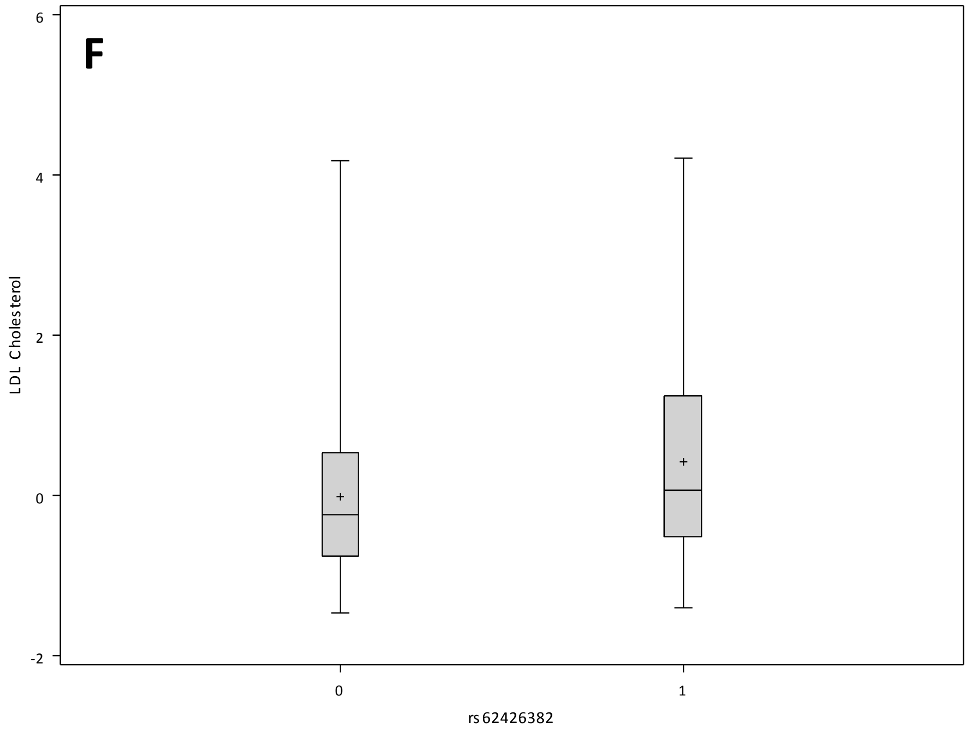

Supplementary Fig 1. Boxplots of traits vs candidate dosage of ref allele (Continued)

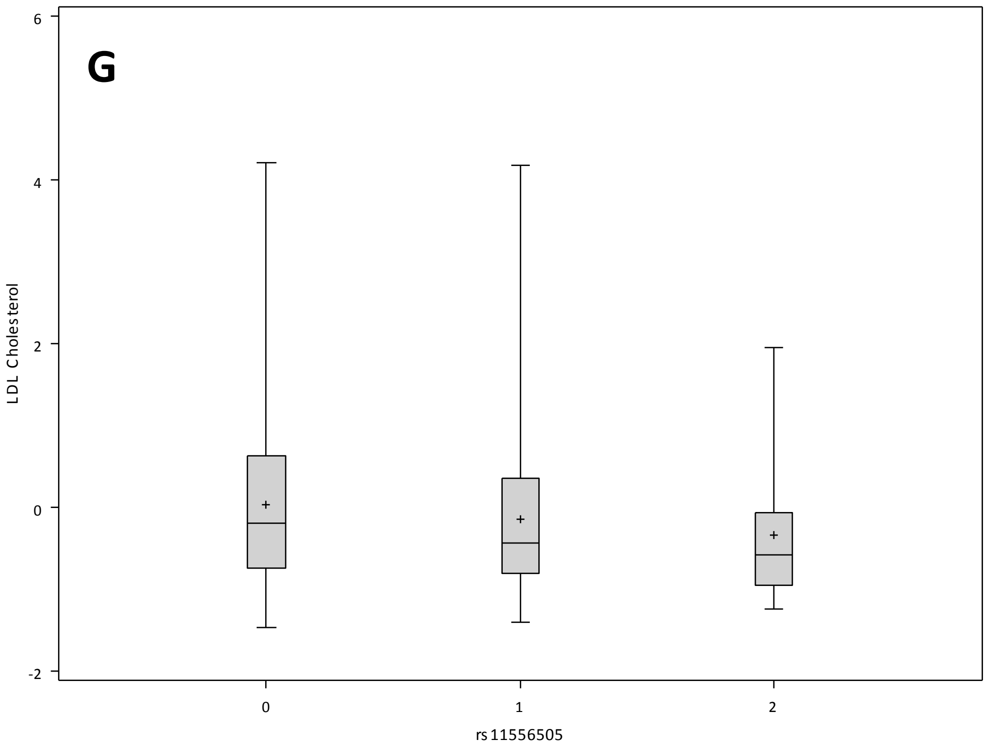

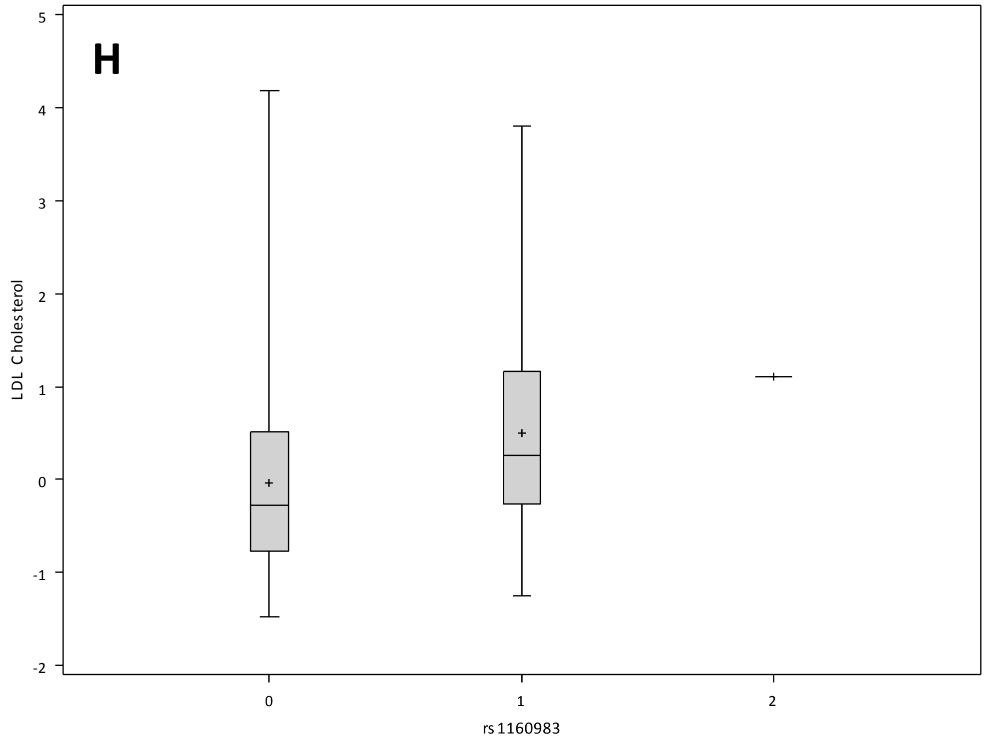

Supplementary Figure 1. Boxplots of traits vs candidate dosage of ref allele (Continued)

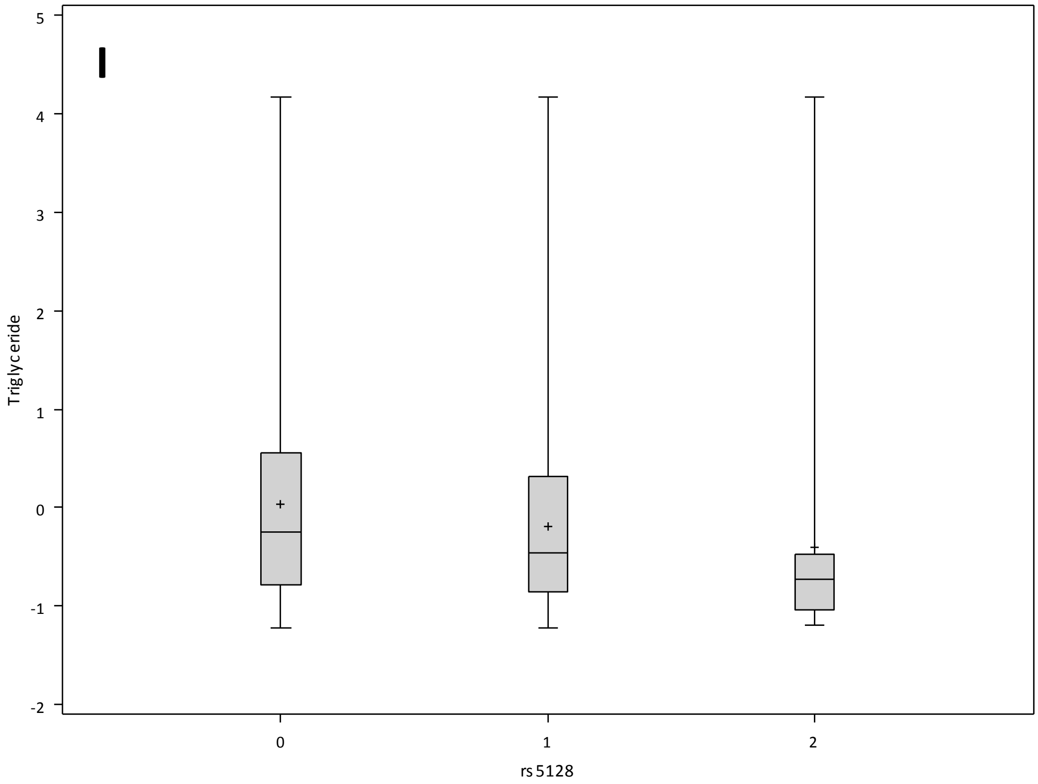

Supplementary Table 7. Published single variants for healthy aging-related genes. A listing of single variants found to have a P-value <0.05 and more than 10 allele copies for “Survival Trait Exceptionality Score” in the Long Life Family Study sequencing. These results validate existing reports finding the same variants to be associated with healthy aging. Those reports can be found by referencing the PubMed ID in the far right column. Referenced under *Results: Sample characteristics: Single variant analyses*.

Phenotype: Survival trait exceptionality score													
chr	***base position***	***rsID***	***gene***	***Function***	***Exonic Function***	***Variant***	***Ref allele***	***Variant allele frequency***	***Number of individuals tested***	***Beta***	***Std Error***	***P value***	***Pubmed ID***
1	7827441	rs35705966	CAMTA1	UTR3		A	T	0.236	809	-0.162	0.058	5.70E-03	22279548
1	12304694	rs79820334	VPS13D	intronic		G	A	0.025	806	0.353	0.17	3.83E-02	22279548
2	31596731	rs17395224	XDH	intronic		A	G	0.027	794	-0.326	0.156	3.70E-02	22279548
2	141773397	rs78806080	LRP1B	exonic	synonymous	A	C	0.067	808	0.231	0.097	1.79E-02	19367585
2	141806541	rs142051427	LRP1B	intronic		T	A	0.011	810	0.698	0.221	1.61E-03	19367585
2	216990772	rs28371549	XRCC5	intronic		G	A	0.042	798	-0.314	0.13	1.56E-02	22406557
2	217070248	rs1051677	XRCC5	UTR3		C	T	0.118	809	-0.226	0.081	5.64E-03	22406557
2	217070652	rs6941	XRCC5	UTR3		A	C	0.117	809	-0.226	0.081	5.64E-03	22406557
3	176742501	rs201627324	TBL1XR1	UTR3		A	G	0.015	691	0.484	0.215	2.47E-02	22279548
5	11082863	rs114692065	CTNND2	exonic	synonymous	T	C	0.005	801	0.644	0.259	1.30E-02	22279548
5	52157374	rs2447867	ITGA1	exonic	synonymous	T	C	0.18	803	-0.167	0.067	1.33E-02	22279548
5	115833090	rs148664380	SEMA6A	intronic		A	G	0.009	740	-0.679	0.315	3.13E-02	22279548
5	172378857	rs539936	ERGIC1	UTR3		C	T	0.046	808	0.32	0.118	6.75E-03	22279548
6	32370860	rs75415018	BTNL2	exonic	synonymous	A	G	0.01	810	-0.514	0.26	4.78E-02	22279548
6	109002042	rs4945816	FOXO3	UTR3		C	T	0.302	801	0.117	0.057	4.04E-02	18765803
6	109003321	rs4946936	FOXO3	UTR3		T	C	0.3	804	0.134	0.057	1.96E-02	18765803
6	109003989	rs9400241	FOXO3	UTR3		C	A	0.299	790	0.129	0.058	2.79E-02	18765803
6	109005588	rs111727905	FOXO3	UTR3		G	A	0.311	807	0.118	0.056	3.56E-02	18765803
6	152423128	rs9341077	ESR1	UTR3		C	T	0.038	809	-0.282	0.136	3.87E-02	20819792
6	152466674	rs2747662	SYNE1	exonic	synonymous	C	T	0.324	808	-0.166	0.053	1.72E-03	22279548
6	152469188	rs2252755	SYNE1	exonic	nonsynonymous	G	C	0.321	794	-0.142	0.054	9.57E-03	22279548
6	152469331	rs2252748	SYNE1	exonic	synonymous	T	C	0.274	774	-0.151	0.057	8.73E-03	22279548
6	152640110	rs2306914	SYNE1	exonic	nonsynonymous	A	G	0.023	809	-0.442	0.172	1.02E-02	22279548
6	152646279	rs9383987	SYNE1	exonic	synonymous	C	G	0.025	806	-0.337	0.168	4.51E-02	22279548
6	152784621	rs9397509	SYNE1	exonic	nonsynonymous	C	T	0.007	809	0.584	0.251	2.04E-02	22279548
7	90675195	rs3779581	CDK14	intronic		T	C	0.158	808	0.168	0.068	1.39E-02	22279548
7	90747371	rs3735649	CDK14	intronic		C	T	0.037	805	0.299	0.145	3.91E-02	22279548
7	90838617	rs12534311	CDK14	UTR3		A	G	0.064	809	0.346	0.101	6.43E-04	22279548
7	90838869	rs3814098	CDK14	UTR3		G	C	0.118	806	0.188	0.079	1.74E-02	22279548
7	92234222	rs200127512	CDK6	downstream		A	G	0.461	650	0.259	0.118	2.89E-02	22279548
7	92241262	rs74980371	CDK6	UTR3		T	C	0.042	810	-0.267	0.132	4.35E-02	22279548
7	94946084	rs854560	PON1	exonic	nonsynonymous	T	A	0.361	770	0.154	0.056	6.17E-03	12082503
7	124481185	rs35536751	POT1	exonic	nonsynonymous	A	C	0.019	810	0.493	0.198	1.31E-02	22113349
7	139715641	rs8192868	TBXAS1	exonic	nonsynonymous	A	G	0.019	810	0.528	0.251	3.60E-02	22279548
8	28927901	rs148967534	KIF13B	UTR3		T	A	0.015	541	-0.611	0.284	3.20E-02	22279548
8	28950309	rs17526980	KIF13B	exonic	nonsynonymous	T	C	0.02	789	0.512	0.218	1.89E-02	22279548
8	113241088	rs1592624	CSMD3	exonic	nonsynonymous	T	G	0.453	504	0.118	0.059	4.80E-02	22279548
9	74319576	rs25696	TMEM2	exonic	synonymous	T	A	0.027	808	0.326	0.154	3.46E-02	22279548
9	74319677	rs17057133	TMEM2	exonic	nonsynonymous	C	T	0.024	808	0.446	0.162	5.92E-03	22279548
9	74360209	rs373799377	TMEM2	exonic	synonymous	C	G	0.133	809	0.169	0.077	2.86E-02	22279548
9	74360209	rs3739783	TMEM2	exonic	synonymous	C	G	0.133	809	0.169	0.077	2.86E-02	22279548
9	129459181	rs71497630	LMX1B	UTR3		T	C	0.06	612	-0.27	0.11	1.43E-02	22279548
10	1228575	rs76022202	ADARB2	UTR3		A	G	0.013	809	-0.608	0.238	1.09E-02	20011587
10	67726514	rs2105702	CTNNA3	intronic		C	A	0.292	810	0.114	0.054	3.73E-02	22279548
10	68040240	rs10997034	CTNNA3	exonic	synonymous	T	G	0.029	810	0.271	0.132	4.13E-02	22279548
10	131633911	rs74562419	EBF3	UTR3		A	G	0.011	810	0.558	0.259	3.14E-02	22279548
11	216290	rs10714	SIRT3	UTR3		A	G	0.033	810	0.323	0.136	1.80E-02	14580859
11	16809573	rs1557875	PLEKHA7	UTR3		A	G	0.169	808	0.154	0.068	2.31E-02	22279548
11	115045674	rs117706029	CADM1	UTR3		A	G	0.009	810	-0.694	0.327	3.43E-02	22279548
11	116703640	rs5128	APOC3	UTR3		G	C	0.093	797	-0.204	0.089	2.21E-02	8018664
12	121439598	rs11065390	HNF1A	UTR3		A	G	0.015	798	-0.551	0.232	1.77E-02	20834067
12	132406666	rs7138581	ULK1	UTR3		C	G	0.107	800	-0.17	0.08	3.44E-02	23061800
13	43787647	rs1037022	ENOX1	downstream		C	T	0.364	771	0.164	0.054	2.64E-03	22279548
13	43788036	rs703206	ENOX1	UTR3		G	T	0.388	807	0.103	0.052	5.00E-02	22279548
13	95058076	rs113980048	GPC6	UTR3		A	C	0.021	810	0.419	0.197	3.40E-02	22279548
13	100425074	rs74694961	CLYBL	intronic		T	G	0.012	810	0.656	0.233	4.93E-03	22279548
13	110407815	rs2289047	IRS2	UTR3		A	C	0.318	806	-0.118	0.055	3.07E-02	19887537
13	110408312	rs139324222	IRS2	UTR3		T	G	0.027	810	0.492	0.175	5.00E-03	19887537
14	37203697	rs10135196	SLC25A21	intronic		A	G	0.232	809	0.124	0.062	4.71E-02	22279548
14	77254836	rs2242624	ANGEL1	UTR3		A	C	0.291	810	0.121	0.056	3.23E-02	22279548
14	81422178	rs2234919	TSHR	exonic	nonsynonymous	A	C	0.043	666	0.254	0.112	2.40E-02	19837933
15	99500605	rs17847203	IGF1R	exonic	synonymous	T	C	0.032	789	-0.373	0.149	1.25E-02	12843179
15	99501545	rs34875596	IGF1R	UTR3		T	C	0.027	759	-0.453	0.155	3.54E-03	12843179
15	99502527	rs1058696	IGF1R	UTR3		T	C	0.028	773	-0.362	0.155	1.93E-02	12843179
15	99503836	rs9282715	IGF1R	UTR3		C	T	0.053	723	-0.348	0.107	1.14E-03	12843179
15	99504004	rs61736167	IGF1R	UTR3		A	G	0.036	808	-0.365	0.128	4.35E-03	12843179
16	50251590	rs3887556	PAPD5	intronic		G	A	0.311	798	-0.123	0.055	2.61E-02	22279548
16	50265818	rs4611456	PAPD5	UTR3		C	T	0.226	807	-0.123	0.058	3.44E-02	22279548
16	50265862	rs4374170	PAPD5	UTR3		G	T	0.227	805	-0.124	0.059	3.47E-02	22279548
16	57007353	rs5883	CETP	exonic	synonymous	T	C	0.069	808	0.207	0.098	3.41E-02	14559957
17	33445549	rs9901455	RAD51D	exonic	synonymous	A	G	0.098	809	0.253	0.082	2.16E-03	22279548
17	78166385	rs11658460	CARD14	exonic	synonymous	T	C	0.061	668	-0.28	0.124	2.40E-02	22279548
19	7114247	rs139376149	INSR	UTR3		T	C	0.004	810	0.666	0.328	4.28E-02	15582274
19	7166388	rs2229429	INSR	exonic	synonymous	A	G	0.194	810	0.163	0.064	1.14E-02	15582274
19	18475285	rs4808787	PGPEP1	UTR3		G	A	0.34	678	-0.141	0.057	1.40E-02	22279548
19	45396144	rs11556505	TOMM40	exonic	synonymous	T	C	0.101	808	-0.2	0.097	4.07E-02	21418511

Supplementary Table 8. Published genes associated with healthy aging and found to suggestive by burden testing in 810 LLFS participants. A listing of genes found to have a P-value <0.05 by at least one of four burden-testing algorithms. Published reports can be found by referencing the PubMed ID in the far right column. Referenced under *Results: Sample characteristics: Analysis of multiple variants across genes*.

Gene	*RefSeq*	*Gene Exon N*	*Exons Adequately Covered by Sequencing*	*Variants Called N*	*Ped N*	*P-value (UWSS)*	*P-value (WSS)*	*P-value (PWST)*	*P-value (Skat)*	*PUBMED ID*
ACE	NM_000789	25	15	23	47	4.51E-01	9.00E-01	4.20E-02	5.27E-02	8136829
CETP	NM_000078	16	7	10	15	2.61E-02	1.87E-02	2.50E-01	2.79E-01	14559957
CSMD3	NM_198123	71	36	67	118	6.68E-01	4.53E-01	2.90E-01	3.20E-02	22279548
DEFB1	NM_005218	2	2	13	24	7.92E-02	4.19E-02	6.50E-02	2.43E-02	22279548
DOCK2	NM_004946	52	17	29	36	1.19E-01	1.70E-01	6.70E-02	9.20E-03	22279548
FOXO1	NM_002015	3	2	36	70	2.45E-01	6.26E-01	8.80E-02	2.71E-02	19793722
GATA4	NM_002052	7	4	34	61	5.85E-02	9.08E-02	4.20E-02	1.48E-02	22279548
LINGO2	NM_152570	7	3	15	24	2.17E-02	1.87E-02	8.70E-01	8.28E-01	22279548
LMNA	NM_001282625	13	1	6	10	8.66E-01	8.95E-01	1.30E-01	3.08E-02	22279548
LMX1B	NM_002316	8	1	27	37	2.94E-01	3.07E-01	2.80E-01	3.70E-02	22279548
NFKB1	NM_003998	24	9	18	48	2.37E-02	2.09E-02	3.70E-02	4.79E-02	22279548
PAPD5	NM_001040285	13	4	37	55	3.73E-02	3.21E-02	5.40E-02	2.45E-02	22279548
PARK2	NM_004562	12	9	33	52	6.61E-02	3.67E-02	3.50E-01	6.87E-01	22279548
PON1	NM_000446	9	6	13	26	5.31E-01	4.16E-01	9.00E-03	4.34E-02	12082503
PREX1	NM_020820	40	8	12	19	7.18E-01	6.12E-01	1.01E-01	2.50E-02	22279548
RAD51D	NR_037711	9	2	16	32	5.27E-02	9.41E-02	6.90E-02	4.10E-02	22279548
SIRT3	NM_012239	7	4	15	25	9.54E-01	8.21E-01	1.30E-01	4.22E-02	14580859
SLC6A4	NM_001045	15	5	17	30	1.11E-01	9.33E-03	3.70E-02	3.34E-02	16095668
TOMM40	NM_006114	10	1	6	12	8.48E-01	7.72E-01	2.00E-02	9.42E-02	21418511
WDR72	NM_182758	20	9	42	61	1.80E-01	2.03E-01	3.50E-01	3.53E-02	20834067

References:

Wu, J., et al. (2005). "A summary of the effects of antihypertensive medications on measured blood pressure." Am J Hypertens **18**(7): 935-942.

Wu, J., et al. (2007). "An investigation of the effects of lipid-lowering medications: genome-wide linkage analysis of lipids in the HyperGEN study." BMC Genet **8**: 60.
